# Assessing life-space mobility for a more holistic view on wellbeing in geriatric research and clinical practice

**DOI:** 10.1007/s40520-018-0999-5

**Published:** 2018-08-04

**Authors:** Joanne K. Taylor, Iain E. Buchan, Sabine N. van der Veer

**Affiliations:** 1 Health e-Research Centre, Farr Institute for Health Informatics Research, The University of Manchester, Vaughan House, Portsmouth Street, M13 9GB Manchester, UK; 2 Manchester University Hospitals NHS Foundation Trust, Manchester, UK; 3 Centre for Health Informatics, Division of Informatics, Imaging & Data Sciences, University of Manchester, Manchester, UK

**Keywords:** Aged, Life space, Mobility, Social isolation, Activities of daily living

## Abstract

Life-space mobility (LSM) is a holistic measure of resilience to physical decline and social isolation in later life. To promote its use as an outcome in geriatric studies and in clinical practice, this review paper explains the concept of LSM; outlines available questionnaires for LSM assessment, provides an overview of associations between LSM and other outcomes, and discusses emerging methods to measure LSM using wearable sensors. Based on performed activity around a central geographical anchor, LSM aims to quantify the observed contraction of daily activities associated with ageing. Several questionnaires are available to assess LSM in different contexts: the University of Alabama Life-Space Assessment and the Life-Space Questionnaire (community settings), the Nursing Home Life-Space Diameter (nursing home settings) and Life Space at Home (for house-bound populations). Some studies using GPS trackers to calculate life-space parameters reported promising results. Although these techniques reduce data collection burden, battery life and older people’s willingness to wear a tracker require further improvement before they can be used more widely. Regardless of the assessment method used, LSM was associated with measures of functional and cognitive abilities, nursing home admission and mortality. The current availability of instruments, the ongoing development of less burdensome data collection techniques, and evidence of construct validity support a case for promoting integration of LSM assessments into geriatric research studies and clinical practice. Ultimately, this will provide a more holistic view on older people’s health and wellbeing.

## What is life-space mobility?

The maintenance of an active lifestyle and social participation in older age is fundamental for overall wellbeing. Numerous constructs may assess certain aspects of ‘successful ageing’ in isolation, such as preservation of physical functioning, absence of age-related disability and self-reported satisfaction [1].

Life-space mobility (LSM) aims to provide a more holistic measure of resilience to physical decline and social isolation in later life [2], with life space describing the physical and social environment a person inhabits on a day-to-day basis. Life space is commonly structured into various ‘life zones’, centred around a central geographical anchor. For example, the bedroom may be the central zone, moving outwards into the rest of the house, the building perimeter, local community, neighbourhood, or town. LSM is graded on a numerical scale and reflects the way in which a person moves across life-space zones over a given time period, while incorporating the frequency and independence (i.e. requirement for assistance/mobility aids) of these movements.

LSM focuses on the performed actions rather than abilities of a person. This distinguishes it from traditional measures such as the ‘get up and go’ test or gait speed, which tend to focus on physiological reserve and functional capacity [3]. It provides a more complete picture of what a person ‘does do’ rather than what they physically ‘can do’. For example, a severely disabled person may utilise a mobility aid and accessible transport to maintain a large life space, whilst a physically able person with dementia or depression may be relatively restricted.

Based on a comprehensive review of the literature, Webber et al. [4] proposed a theoretical framework of mobility in older adults. LSM forms a central element in their framework, alongside a wide range of factors that may affect mobility, such as financial and environmental influences. Furthermore, measuring LSM has the potential to provide valuable information on mobility and social participation in older age, for example, to identify ‘age friendly’ needs within a local area, or to act as a measure of effectiveness of interventions targeted at older people. Lastly, as LSM facilitates a more holistic approach to a person’s physical status, it aligns with older people’s preferences for a broader perspective on health care outcomes, which includes improved functioning and reduced dependency [5].

To further promote the use of LSM as an outcome in geriatric studies and clinical practice, this review paper aims to provide an overview of available questionnaires to assess LSM. We searched the available literature using the search term ‘life-space’ on the following electronic databases: The Cochrane Central Register of Controlled Trials (CENTRAL), Medline, Embase, The Cumulative Index to Nursing and Allied Health Literature (CINAHL) and Clinicaltrials.gov. (initial search October 2016, update in April 2018). All studies measuring LSM where the majority of participants were aged > 65 were included. As well as summarising commonly used assessment techniques, we also reviewed the associations between LSM and other outcomes. Lastly, we discuss emerging methods to measure LSM using wearable and sensor technologies.

## Questionnaires to assess LSM

Our literature search yielded four questionnaires for assessing LSM (see Table 1), which we discuss in more detail below.

**Table 1:** Available questionnaires to assess LSM and their characteristics

Questionnaire	Population	Administration	Format	Aspects of LSM measured	Timeframe	Score	Examples of studies that used the questionnaires
University of Alabama Life-Space Assessment (UAB-LSA) [6]	Community dwelling older people	Interviewer, self-report	9 questions Graded responses	Area: five zones Frequency: yes Independence: yes	4 weeks	Composite score based on three domains, 0–120	MrOS [7] SAOS [8] E-SAS [9, 10] UAB [6, 11, 12] LISPE [13–15] IMIAS [16]
Life-space Questionnaire (LSQ) [17]	Community dwelling older people	Interviewer, self-report^a^	9 questions Yes/no responses	Area: nine zones Frequency: no^b^ Independence: no^b^	3 days^c^	Simple tally score, 0–9	ACTIVE [18–20] WHAS-1^d^ [21] MAP^d^ [22, 23] MARS^d^ [23, 24]
Nursing Home Life-Space Diameter (NHLSD) [25]	Nursing home residents	Interviewer	4 questions Graded responses	Area: four zones Frequency: yes Independence: yes	2 weeks	Total score = multiplication of three domains, 0–100	Tinetti and Ginter 1990 [25] Nordic RCT [26, 27] REDICNH [28]
Life-Space at Home (LSH) [29]	Housebound older people	Interviewer	A set of instructions for score calculation	Area: four zones Frequency: yes Independence: yes	1 week	Total score = multiplication of three domains, unlimited score	Hashidate et al. [29]

*MrOS* The Osteoporotic Fractures in Men Study, *SAOS* The South Australian Omnibus Survey, *E-SAS* The Elderly Status Assessment Set, *UAB* University of Alabama at Birmingham Study of Ageing, *LISPE* Life-Space Mobility in Old Age Study, *IMIAS* International Mobility in Aging Study, *ACTIVE* Advanced Cognitive Training for Independent and Vital Elderly, *WHAS-1* The Women’s Health and Aging Study I, *MAP* Rush Memory and Aging Project, *MARS* The Minority Aging Research Study, *Nordic RCT* Nordic multi-centre study on physical and daily activities for residents in nursing home settings, *REDNICNH* Recourse Use and Disease Course in dementia—Nursing Home study

^a^Self-report used with modified versions only

^b^Some modified versions have adapted to include this

^c^Some modified versions have expanded this

^d^Modified version used

### University of Alabama Life-Space Assessment (UAB-LSA)

The UAB-LSA is by far the most common LSM assessment method [6]. Baker and colleagues set out the original validation for the composite UAB-LSA score using longitudinal data from the prospective University of Alabama at Birmingham (UAB) Study of Aging [6]. Their study population represented a random sample of 306 Medicare beneficiaries aged 65 or over. Test–retest reliability was 0.96 [95% Confidence Interval (CI), 0.95–0.97] when comparing composite UAB-LSA scores at initial interview to 2-week follow-up phone call.

Although the questionnaire was designed to be interviewer administered, some studies showed the questionnaire’s suitability for self-report. For example, the South Australian Omnibus postal survey [8] included the UAB-LSA for self-report, achieving an overall response rate of 59.5%.

The questionnaire distinguishes five life-space levels: (1) other rooms than the bedroom, (2) area outside the house, (3) neighbourhood, (4) outside neighbourhood, but in town, and (5) outside town. Each life-space level is allocated a sub-score based on a multiplication of average weekly frequency and independence of movement (based on preceding 4 weeks), and a total composite score is summated across levels, with a maximum of 120.

Mean composite scores varied by studied population. The largest prospective study of LSM to date found a mean score of 84.9 [standard deviation (SD) 24.2] [7]. This was a cohort of 3892 relatively healthy, community dwelling men, aged 71–98 years. Other studies have reported significantly lower mean scores varying from 41.7 (SD 20.9) to 64.5 (SD 24.9) [11, 30]. Some studies used a composite UAB-LSA score < 60 to define ‘restricted LSM’ [11, 13].

The UAB-LSA has been tested in at least 13 different languages in older, community-dwelling populations across the world.

### The Life-Space Questionnaire (LSQ)

The original LSQ was developed by Stalvey et al. [17]. For the original validation study, they recruited 200 people with cataracts aged 55–85 from an outpatient eye clinic. They found LSQ scores were positively skewed. This is likely due to the relatively young cohort. In addition, the questionnaires were administered in clinic, thus likely excluded many patients unable to travel independently to the hospital.

The LSQ was designed to be interviewer administrated, but has also been used for self-completion, with response rates ranging from 67 to 85% [22, 31]. Use of LSQ in participant completed surveys appeared to be acceptable and feasible.

The questionnaire comprises nine ‘yes/no’ questions regarding a person’s movements across nine life-space zones in the preceding 3 days (questionnaire available online at: https://www.uab.edu/medicine/ophthalmology/images/research/LifeSpace.pdf). The first six zones are similar to the levels in the UAB-LSA, with level 2 (area outside the house) split into two zones: immediately outside your home, e.g. porch, and further outside your home perimeter, e.g. parking lot. The last three zones refer to (7) outside your county, (8) outside the state and (9) outside the region. The total score ranges from 0 to 9, reflecting the number of zones a person has moved in; the frequency and independence of these movements are not taken into account. Several studies have modified the original LSQ. For example, by expanding the questionnaire to include the preceding 2 months [18], by assessing six life-space zones in the last week [22], using a four-point scale for only two zones [21], or using a reversed scale of 0–5, with 0 implying high LSM [24].

For the original LSQ instrument, scores tend to be skewed towards the positive end of the scale. Some studies found a mean score of 6 (SD 1.4) in a community-dwelling older population [17, 31], whereas Satori et al. reported a mean LSQ score of 7 (SD, 1.3) [18]. The latter studied a population of 2,737 participants who lived largely independent of formal care. Stalvey et al. [17] suggested to define a restricted LSM as a total score of 5 or lower. This was accepted by Byles et al. [31], who further suggested classifying a score of 6 as ‘mid’ and of 7–9 as ‘high’ scores.

### The Nursing Home Life-Space Diameter (NHLSD) questionnaire

The NHLSD questionnaire was developed by Tinetti et al. in 1990 [25]. It was validated in 398 residents of three nursing homes in New Haven, Connecticut [25]. Average subject age was 82 (range 69–93), and residents who were bedbound, chair-bound or ‘restrained’ were excluded. Test–retest reliability was high (*r* = 0.92), as was reliability between assessors (*r* = 0.95). The NHSLD was designed to be nurse-administered only.

The score involves a calculation based on the frequency of residents’ movement, ranging from 0 (bedbound) to 50 (leaving the facility daily) based on four life-space thresholds: within residents’ own room (1), within the unit (2), outside the unit (3), and outside the facility (4). Scores can be multiplied by 2 to indicate complete independence of movement, resulting in a maximum score of 100. However, the majority of scores tend to be in the lower end of the 0–100 range. Tinetti et al. [25] found a mean NHLSD score of 27.1 (SD 10.2) among 398 residents. Bergland et al. [26] reported a mean score of 25.2 (SD 10.9) in 322 nursing home residents in Scandinavia who required assistance with activities of daily life. The Recourse Use and Disease Course in dementia—Nursing Home (REDICNH) study looked at 696 new NH admissions across Norway and found a median score of 36 (interquartile range 26).

When performing LSM assessments in an institutionalised environment, mobility is more likely to be influenced by external factors such as staff availability and facility routine, rather than being primarily driven by intrinsic motivation of the participant. Whilst this still conforms to the mobility construct as set out by Webber et al. [4], direct comparison with community-based LSM assessments should be made with caution.

### Life Space at Home (LSH) assessment

The LSH assessment was designed to measure LSM for housebound older people only. It was developed and validated by Hashidate et al. [29], who included 20 housebound community-dwelling older people undergoing a home-care rehabilitation programme. It was designed to be administered by researchers. Since its development in 2013, deployment of the instrument in other studies has not been reported.

For the assessment, four unique life-space ‘destinations’ within the participant’s home are defined (for example, entrance, dining room, bathroom, and toilet), and a measurement of distance between each destination and the participant’s bedroom is taken. This makes the assessment relatively complex, and may limit the instrument’s use outside a research context. For each destination, a score is then calculated by multiplying this distance by 2 if performed independently, or by 1.5 if using equipment; and then again multiplied by the frequency of movements in the preceding week. The final LSH score is the summation of these four destination-specific scores. As each participant’s score is unique based on the size of their house, there is no upper limit for the total score. Hashidate et al. [29] reported a mean LSH score across all 20 participants of 643.2 (SD 461.1).

As with the NHLSD questionnaire, scores will be influenced by the availability of assistive services, thus LSM score will be more dependent on external factors than in regular community settings.

## Novel methods to assess LSM

Using questionnaires to measure LSM requires a researcher or health care professional to perform the assessment, or instead relies on self-report by older people. This data collection burden may partly explain why LSM questionnaires are not widely used in clinical practice. Sensor technologies enable the unobtrusive collection of objective, longitudinal data. Several studies have used accelerometers to measure physical activity alongside questionnaire-based LSM assessments [32–34]. Assessing LSM, however, requires not just measurement of activity, but also movements across life-space zones. Using sensor technology in isolation, this can only currently be achieved using Global Positioning System (GPS) technology for community-dwelling older people. Most smartphones have a built-in GPS and are becoming more ubiquitous every day, therefore, offering promise to become a feasible alternative to tradition self-report methods.

GPS uses a person’s geolocation to calculate various measures related to LSM, such as number of trips away from home, maximum distance travelled away from home and total area travelled (sometimes termed ‘life-space’). The travel mode (walking, car, and public transport) for each trip can be determined based on speed and place of travel.

Studies so far have shown it is possible to use GPS trackers for calculating life-space parameters [35–38] while others showed promising results regarding acceptability and feasibility of this method in an older population [39, 40]. Yet, these aspects may need continued attention to minimise the amount of missing data. Tung et al. piloted the use of GPS trackers to compare LSM between people with Alzheimer’s disease (AD) and controls (*n* = 33) [41]. They reported a mean wear time of only 7.46 h/day (SD 2.01) despite participants being asked to wear the device from awaking to bedtime. In addition to participants’ non-compliance to the study protocol, they also identified poor battery life as an issue. Another study in 279 functionally and cognitively independent older people in retirement communities found an average wear time of 13.6 h/day (SD 1.3) [37]. The ‘Mobility Study’, which recruited older people to wear GPS trackers for 1 week had to exclude 15 of 86 participants due to missing sensor data [42]. Lastly, Liddle et al. [38] reported problems with GPS measurement accuracy within the home environment.

Sensor networks are another related technology used to measure area and frequency of movements, especially in and around a person’s residence. Still in its early stages, Jansen et al. [43] have published a study protocol for implementing a wireless sensor network within a nursing home environment. Participants will wear body-worn sensors that communicate with a network of anchor nodes to continuously monitor people’s location. Once validated, this new method could provide a sensor-based equivalent of the NHLSD or LSH questionnaires [25, 29].

Table 2 summarises the advantages and disadvantages of each LSM assessment technique presented.

**Table 2:** Advantage and disadvantages of available life-space mobility (LSM) assessment methods

Assessment method	Advantages	Disadvantages
Questionnaires		
University of Alabama Life-Space Assessment (UAB-LSA) [6]	Well-established and commonly used method Very strong evidence base for validity Suitable for self-report Takes into account multiple dimensions of LSM (i.e. area, frequency, and independence of movement)	High-data collection burden (minimum 20 items) Only validated in community-dwelling populations Requires calculation of composite score
Life-space Questionnaire (LSQ) [17]	Well-established and commonly used method Strong evidence base for validity Low-data collection burden Suitable for self-report	Only takes into account one dimension of LSM (i.e. area) Only validated in community-dwelling populations
Nursing Home Life-Space Diameter (NHLSD) [25]	Moderate evidence base for validity Takes into account multiple dimensions of LSM (i.e. area, frequency, and independence of movement)	Only validated in nursing home populations Not suitable for self-report LSM scores vulnerable to influences by external factors Not fully aligned with theoretical construct^a^ Requires calculation of composite score
Life Space at Home (LSH) [29]	Moderate evidence base for validity Takes into account multiple dimensions of LSM (i.e. area, frequency, and independence of movement)	Not commonly used Only validated in housebound populations Not suitable for self-report Practically challenging and laborious to perform (requires measuring distances in participant’s home) Requires calculation of composite score
Sensor methods		
GPS trackers [35–42]	Low-data collection burden Objective data on LSM (decreases risk of bias)	Limited evidence base for validity Requires participants’ engagement (e.g. to wear and recharge the tracker) Requires data processing algorithms to derive LSM metrics from raw tracker data Unclear what are meaningful GPS-derived LSM metrics Ethical considerations associated with remote monitoring
Residence-based sensor networks [43]	Very low data collection burden Requires no active engagement from participants Objective data on LSM (decreases risk of bias)	No evidence base for validity Only applicable to housebound/residential housed populations Unclear what are meaningful network-derived LSM metrics Costs associated with installing, monitoring and maintaining network systems Ethical considerations associated with remote monitoring

*GPS* global positioning system, *LSM* life-space mobility

^a^Mobility construct as described by Webber et al. [4] assumes degree of individual autonomy—may not be applicable for most dependent residents

## Associations between LSM and other outcomes

This section provides an overview of LSM’s construct validity [44] by discussing the association between LSM scores and more traditional outcomes, as reported by the studies identified in our search. Other potentially relevant outcomes for older people, so-called ‘geriatric syndromes’ such as polypharmacy, nutrition and incontinence have yet to be investigated.

### Physical functioning

UAB-LSA and LSQ scores were significantly correlated with the physical component score of the SF12 and SF36, respectively [6, 31]. The UAB-LSA strongly correlated with physical assessments, such as the timed get-up-and-go test, short physical performance battery and gait speed as well as functional measures such as independence with ADLs in various studies [6, 7, 45, 46]. Bergland et al. [26] found a positive correlation between NHLSD and LSH scores and several markers of physical capacity, such as muscle strength (*r* = 0.59; *P* = 0.01), grip strength (*r* = 0.35; *P* < 0.001), walking speed (*r* 0.33; *P* < 0.001) and the timed get-up-and-go test (*r* = − 0.74; *P* = 0.01). Similarly, Takemoto et al. showed that the number, time and distance of GPS-measured pedestrian trips were significantly related with the 400-m walk test and physical performance battery [37]. Lastly, several studies reported associations between LSM scores and (the need for assistance with) activities of daily living [6, 24, 25].

### Psychological and cognitive functioning

Crowe and colleagues reported that baseline UAB-LSA scores predicted cognitive decline over a 4-year follow-up (adjusted difference = − 0.177; *P* < 0.001) [12]. Similar reports were found using the LSQ [23]. Tung et al. and Giannouli et al. reported an association between diagnosis of Alzheimer’s dementia/cognition and GPS measures of life space [41, 42]. Temporal analysis from 2-year follow-up data from the LISPE study in Finland suggested executive function was a determinant of life-space mobility [47]. Takemoto et al. showed that the number and distance of GPS-measured pedestrian trips correlated with depression and fear of falling [38]. However, there were no relations with measures of cognitive functioning. In the same study, vehicle trips did not correlate with functioning, apart from an observed negative correlation with fear of falling (*r* = − 0.89; *P* < 0.05).

### Falls and healthcare utilisation

Lo et al. described a significant correlation between LSM and incident falls on multivariate analysis during 6-month follow-up from the UAB Study of Ageing (OR for each ten-point decrement in life space = 1.16, 95% CI 1.03–1.31, *P* = 0.02) [48]. Follow-up at 36 months found an association between a decline in UAB LSA score and healthcare utilisation (as measured by self-reported ED visits and hospitalisation episodes) [49].

### Mortality and nursing home admission

Boyle et al. combined data for 1,445 older participants over up to 8 years (mean follow-up, 4.1 years). After adjustment for multiple variables, people with restricted LSM (as measured by LSQ) carried a greater risk of death compared to those without restriction [adjusted hazard ratio (HR) 1.18, 95% confidence Interval (CI) 1.09–1.27] [24]. Similarly, another study demonstrated that lower composite UAB-LSA scores were associated with a higher risk of non-cancer mortality over 2.7-year follow-up (adjusted HR 3.82, 95% CI 1.27–11.53) [7]. This was confirmed by Mackey et al., who additionally found that participants with restricted LSM at baseline had a greater risk of nursing home admission over the 6-year follow-up period (11.9 vs. 2.7%; *P* < 0.001) [2].

## In summary

The current availability of LSM measurement instruments, the ongoing development of GPS-based techniques for less intrusive data collection, and evidence of associations between LSM and a range of traditional outcomes support a case for promoting integration of LSM assessments into geriatric research studies and clinical practice. Future research should focus on developing cheap, reliable sensor or smartphone-based LSM methods. This seems the natural progression to facilitate collection of relatively large volume, complex data, and opens up the possibility to offer viable platforms for digital interventional studies. Not only would this offer the potential to develop software capable of creating personalised life-space maps based on individual’s ‘baseline’ movements and surrounding area, but could offer a viable platform for digital interventional studies. Ultimately, this will help health care professionals and policy-makers to establish a more holistic view on older people’s health and wellbeing.
